# Predictive value of angiogenesis-related gene profiling in patients with HER2-negative metastatic breast cancer treated with bevacizumab and weekly paclitaxel

**DOI:** 10.18632/oncotarget.8128

**Published:** 2016-03-16

**Authors:** Marta Mendiola, Virginia Martínez-Marin, Jesús Herranz, Victoria Heredia, Laura Yébenes, Pilar Zamora, Beatriz Castelo, Álvaro Pinto, María Miguel, Esther Díaz, Angelo Gámez, Juan Ángel Fresno, Ana Ramírez de Molina, David Hardisson, Enrique Espinosa, Andrés Redondo

**Affiliations:** ^1^ Molecular Pathology and Therapeutic Targets Group, La Paz University Hospital – IdiPAZ, Madrid, Spain; ^2^ Department of Medical Oncology, La Paz University Hospital, Madrid, Spain; ^3^ Translational Oncology Group, La Paz University Hospital – IdiPAZ, Madrid, Spain; ^4^ IMDEA, Campus de Excelencia Internacional CEI (UAM-CSIC), Madrid, Spain; ^5^ Department of Pathology, La Paz University Hospital, Madrid, Spain

**Keywords:** metastatic breast carcinoma, bevacizumab and weekly paclitaxel, predictive, angiogenesis, gene expression

## Abstract

Bevacizumab plus weekly paclitaxel improves progression-free survival (PFS) in HER2-negative metastatic breast cancer (mBC), but its use has been questioned due to the absence of a predictive biomarker, lack of benefit in overall survival (OS) and increased toxicity. We examined the baseline tumor angiogenic-related gene expression of 60 patients with mBC with the aim of finding a signature that predicts benefit from this drug.

Multivariate analysis by Lasso-penalized Cox regression generated two predictive models: one, named G-model, including 11 genes, and the other one, named GC-model, including 13 genes plus 5 clinical covariates. Both models identified patients with improved PFS (HR (Hazard Ratio) 2.57 and 4.04, respectively) and OS (HR 3.29 and 3.43, respectively). The G-model distinguished low and high risk patients in the first 6 months, whereas the GC-model maintained significance over time.

## INTRODUCTION

Breast cancer is a heterogeneous disease regarding molecular and clinical features. In the metastatic setting, the expression of human epidermal growth factor receptor 2 (HER2) and hormonal receptors determine the selection of therapies. Treatment for HER-2 negative mBC includes hormonal therapy, chemotherapy and bevacizumab combined regimens.

In the pivotal E2100 study, bevacizumab plus weekly paclitaxel increased overall response rate (50% vs 22%) and progression-free survival (PFS) (median of 11.8 vs 5.9 months) compared to paclitaxel alone [1]. Other phase III trials in mBC also reported a PFS benefit with the addition of bevacizumab to chemotherapy, both in first [2, 3] and second line therapies [4]. However, none of them showed a significant improvement in overall survival (OS), possibly due to the confounding effect of post-progression therapy, lack of statistical power, or treatment crossover. This, along with economic issues, lack of validated biomarkers and increased toxicity has put into question the role of bevacizumab in mBC. Nowadays, the identification of predictive biomarkers for this drug remains a challenge.

Technological improvements in molecular profiling have allowed a better knowledge of breast cancer biology, leading to the development of new tests that help in clinical decision making.

In the present study we analysed a set of 168 genes related to angiogenesis and other processes on a discrete series of patients treated with bevacizumab and weekly paclitaxel. Our aim was to find a molecular signature predicting PFS benefit from this regimen. To date, this is the first report of a biomarker profile with PFS prediction. We selected genes implicated in angiogenesis and other related processes, such as Epithelial to Mesenchymal Transition (EMT) and inflammation, that could have an impact in the response or resistance to bevacizumab [5].

## RESULTS

### Descriptive statistics and univariate analysis

Sixty patients were included and their clinical characteristics are summarized in Table 1. Median age at diagnosis was 54 years (range 33-76). Almost half of the patients had received prior therapy with anthracyclines and taxanes, and 40% had been treated with one or more previous lines of chemotherapy for metastatic disease.

**Table 1:** Patients characteristics

Patients characteristics	N (%)
Estrogen Receptor (ER)	
Positive	49 (81.7)
Negative	11 (18.3)
Progesterone receptor (PR)	
Positive	41 (68.3)
Negative	19 (31.7)
IHQ subtype	
Triple negative	11 (18.3)
ER positive, PR negative	8 (13.3)
ER positive, PR positive	41 (68.3)
Adjuvant chemotherapy	
Yes	46 (76.7)
No	14 (23.3)
Disease-free interval	
≤ 24 months	17 (28.3)
> 24 months or stage IV at diagnosis	43 (71.7)
Number of chemo lines for metastasic disease	
0	36 (60)
>1	24 (40)
Previous chemotherapy	
None (or non antracyclines: e.g. CMF)	15 (25)
Anthracyclines	17 (28.3)
Anthracyclines and taxanes	28 (46.7)
Metastatic locations	
1 or 2	27 (45)
≥ 3 or hepatic involvement	33 (55)

IHQ: inmunohistochemistry CMF: old chemotherapy regimen

Forty-five percent of the patients achieved a partial response and 13% a complete response. Median PFS was 11.4 months (range 0.5-46.1), and median OS was 29 months (range 1.41-44.8). All patients received bevacizumab until progression, but paclitaxel had to be discontinued after 6-8 cycles in 32 patients, mostly due to toxicity. During the bevacizumab continuation phase, 15 patients (53.5% of estrogen receptor -ER- positive patients with continuation treatment) also received hormonal therapy.

Univariate analysis of clinical and pathological variables for PFS is presented in Table 2. Disease-free interval (DFI), ER and metastatic location (3 or more locations or hepatic involvement) were significantly associated with PFS. Summary information for the gene expression measurements as well as the *Hazard Ratio* (HR) for the association between the gene expression values and PFS is presented on Supplementary Table. Nine genes (*CDH11, ESR1, FABP5, FN1, IL8, NOTCH3, PGR, PLAU* and *SLC39A6*) showed adjusted p values <0.05 for association with cancer progression.

**Table 2:** Univariate analysis of clinical variables for progression-free survival

Variable	HR (95% CI)	P value
Age	1.02 (0.99-1.04)	0.18
Disease-free interval (≥24 vs < 24 months)	0.49 (0.27-0.89)	0.02
Estrogen-receptor (positive vs negative)	0.42 (0.21-0.83)	0.02
Progesterone-receptor (positive vs negative)	0.58 (0.32-1.03)	0.07
Adjuvant chemotherapy (yes vs no)	0.69 (0.37-1.27)	0.25
Prior anthracyclines and taxanes (yes vs no)	1.17 (0.63-2.17)	0.62
Prior lines for metastatic disease (≥ 1 vs 0)	1.64 (0.93-2.87)	0.09
Metastatic location (< 3 locations and no liver involvement vs ≥ 3 or hepatic)	2.92 (1.61-5.32)	0.0003
Hormonal therapy*	0.48 (0.2-1.17)	0.11

* Only in subgroup of patients with bevacizumab continuation treatment and ER positive

### Multivariate cox models

A multivariate analysis with five clinical variables (DFI, ER, ML, prior anthracyclines and taxanes, and prior chemotherapy treatment for metastatic disease) was associated with PFS (Table 3). This was defined as the clinical or C-model.

**Table 3:** Clinical multivariate analysis for progression-free survival (C-model)

Variable	HR (CI 95%)	P value
Disease-free interval (≥24 vs < 24 months)	0.74 (0.35-1.59)	0.43
Estrogen-receptor (positive vs negative)	0.41 (0.17-1.03)	0.06
Prior anthracyclines and taxanes (yes vs no)	1.02 (0.52-2.01)	0.94
Prior lines for metastatic disease (≥ 1 vs 0)	1.55 (0.8-3)	0.19
Metastatic location (< 3 locations and no liver involvement vs ≥ 3 or hepatic)	3.07 (1.63-5.8)	0.0005

**Figure 1:**
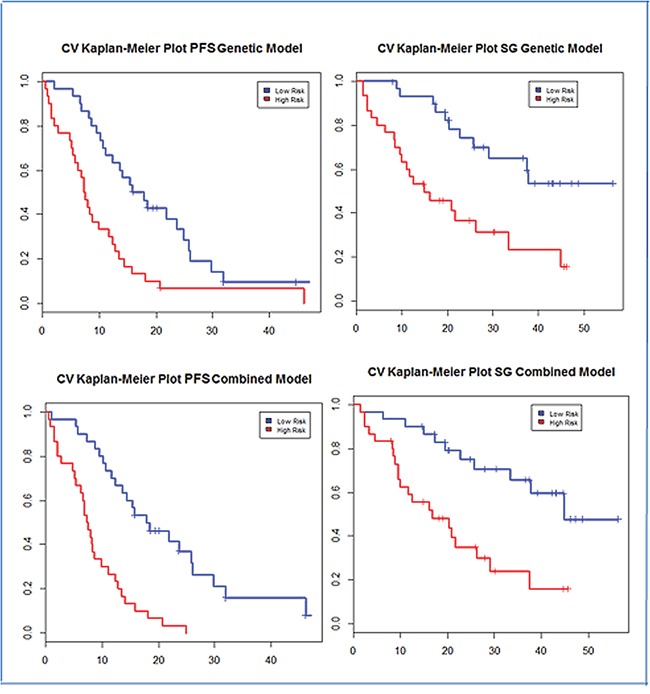
Progression-free survival and overall survival Kaplan-Meier curves of the two groups established by the G-model (a and b) and by the GC-model (c and d)

We then combined clinical and gene variables into two models using the least absolute shrinkage and selection operator (LASSO) [6] [7] for variable selection: one model had gene expression only (G-model), and the other had both gene expression and clinical variables (GC-model). The G-model included 11 genes (*SLC39A6, REL, IL8, FN1, PLAU, HES1, HMBS, DDIT4, FABP5, ACVRL1, PGR*) (Table 4). The GC-model consists of 13 genes (*REL, FN1, NOTCH3, DDIT4, IL8, ADRBK1, FABP5, PLAU, HMBS, PTK2B, THBS1, SLC39A6, TCF3*) and the five clinical variables mentioned previously (Table 5). The beta coefficient signs of the genes agreed with the expression results from the univariate analysis. Figure 1 shows Kaplan Meier plots for progression-free survival (PFS) and overall survival (OS) in G and GC models. The HR, differences in median and p values for these predictions are summarized in Tables 6 (PFS) and 7(OS).

**Table 4:** Genetic model (G-model)

Gene	Beta coefficient (LASSO)	HR (IC95%)
**SLC39A6**	0,289	1,72 (1,28-2,3)
**REL**	−0,282	0,74 (0,56-0,99)
**IL8**	−0,231	0,7 (0,54-0,89)
**FN1**	0,191	1,51 (1,18-1,93)
**PLAU**	0,152	1,5 (1,14-1,98)
**HES1**	0,138	1,15 (0,92-1,44)
**HMBS**	−0,063	0,81 (0,64-1,04)
**DDIT4**	0,049	1,14 (0,87-1,51)
**FABP5**	−0,043	0,72 (0,55-0,96)
**ACVRL1**	0,031	1,32 (0,99-1,77)
**PGR**	0,021	1,46 (1,09-1,95)

**Table 5:** Combined genetic and clinical model (GC-model)

Variable	Beta (LASSO)	HR
Disease-free interval (≥24 vs < 24 months)	−0.223	0.8
Estrogen-receptor (positive vs negative)	−0.907	0.4
Prior anthracyclines and taxanes (yes vs no)	0.008	1.014
Prior lines for metastatic disease (≥ 1 vs 0)	0.45	1.57
Metastatic location (< 3 locations and no liver involvement vs ≥ 3 or hepatic)	1.509	4.52
REL	−0.349	0.71
FN1	0.322	1.38
NOTCH3	0.287	1.33
DDIT4	0.281	1.32
IL8	−0.223	0.8
ADRBK1	−0.21	0.81
FABP5	−0.176	0.84
PLAU	0.159	1.17
HMBS	−0.155	0.86
PTK2B	0.136	1.15
THBS1	0.114	1.12
SLC39A6	0.05	1.05
TCF3	−0.028	0.97

**Table 6:** Hazard ratio, medians of progression-free survival and p-values of the two groups of patients established by the G and GC models

Model	Risk Group	HR (CI 95%)	p-value	Median PFS (months)
**G-model**	Low-risk	1	0,048	16,9 (12,3-25,8)
	High-risk	2.57 (1.47-4.48)		7,4 (5,7-12,4)
**GC-model**	Low-risk	1	<0,001	17,9 (13,7-29,8)
	High-risk	4.04 (2.2-7.44)		7,4 (6,2-11,1)

**Table 7:** Hazard ratio, medians of overall survival and p-values of the two groups of patients established by the G and GC models

Model	Risk Group	HR (CI 95%)	p-value	Median PFS (months)
**G-model**	Low-risk	1	0,001	NR
	High-risk	3.29 (1.57-6.91)		14,9 (9,9-44,8)
**GC-model**	Low-risk	1	<0,001	44,8 (33,3-NR)
	High-risk	3.43 (1.62-7.26)		16,8 (9,9-37,4)

### Comparison of accuracy of prediction of the models through roc curves

**Figure 2:**
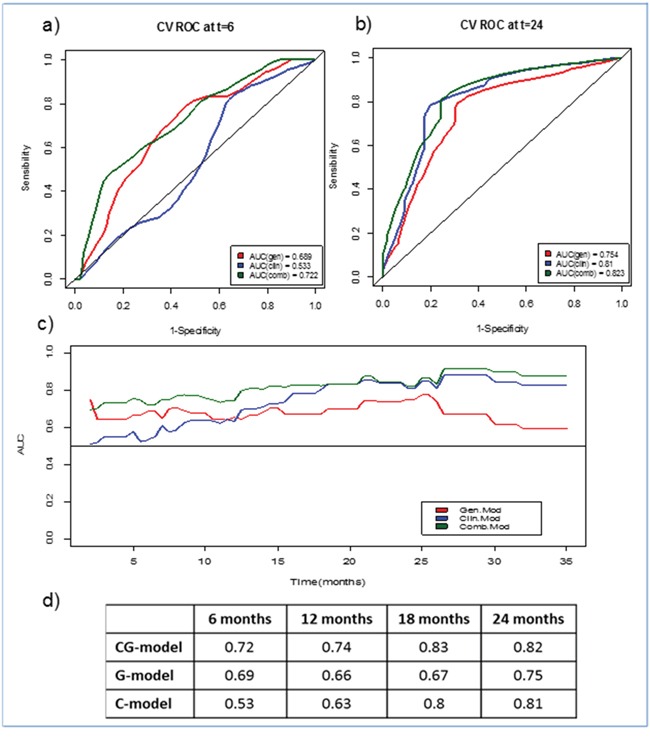
Time-dependent ROC curves for the three models (GC, G and C) **a.** At 6 months, **b.** At 24 months, **c.** AUC evolution for each model over time, **d.** AUC values for each model at four specific time points

Models were also compared to evaluate the gain of accuracy by time-dependent ROC curves [8], and results are shown in Figure 2. In the first 6 months G and GC models worked better than C model (AUC 0.68 and 0.72 versus AUC 0.53 respectively). The G model lose accuracy with longer follow-up, whereas GC remained accurate over time, as shown in Figures 2c and 2d.

### Inmunohistochemistry evaluation of biomarkers

**Figure 3:**
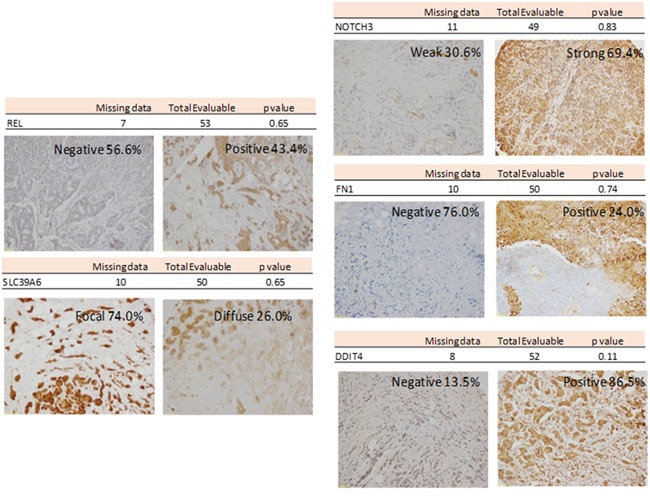
Representative Inmunohistochemistry for REL, SLC39A6, NOTCH3, FN1 and DDIT4

Tissue microarrays (TMA) were used to evaluate the protein expression encoded by those genes with the higher beta coefficients: cREL, SLC39A6, NOTCH, FN1 and DDIT4. A summary of these studies is shown in Figure 3. None of the proteins analyzed were independently associated with PFS.

## DISCUSSION

Bevacizumab was approved as first-line treatment for mBC in 2008. Three years later, the Food and Drug Administration revoked the indication due to increased toxicity without a clear benefit in OS. However, the European Medicines Agency decided to keep the indication, but only in combination with paclitaxel (regimen with the most benefit in SLP).

The identification of a reliable biomarker would avoid unnecessary toxicity in non-responders, reduce healthcare costs, and therefore improve cost-effectiveness. VEGF is a master regulator of angiogenesis, and should be expected to predict response to bevacizumab, but the results of different studies are controversial. Translational studies associated to bevacizumab trials in mBC have shown a possible role of plasmatic VEGFA (pVEGFA) and VEGFR2 levels as response predictors [9-12]. However, preliminary results of the prospective study Meridian showed that baseline pVEGFA levels were not associated to improved PFS [13].

Due to the high number of genes involved in angiogenesis, a single biomarker is unlikely to predict benefit from bevacizumab. Therefore, identification of alternative biomarkers is a main subject for translational oncology research, and, to our knowlegde, this is the first report of a biomarker profile predicting PFS benefit in mBC patients treated with bevacizumab and weekly paclitaxel.

We selected a group of genes described to have a role in angiogenesis and other related processes, to investigate if any combination could impact on response to bevacizumab [5]. In the multivariate analysis we decided to include clinical factors found to be relevant in another study with bevacizumab-containing therapy [14]. Some of these factors did not reach statistical significance, probably due to the small sample size. This is the main weakness of our study, along with the absence of a validation set. Validation was not feasible because of the limited number of patients treated with bevacizumab and paclitaxel. Possible biases are difficult to be controlled in a retrospective study, including the use of chemotherapy before bevacizumab and the use of hormonal therapy during the bevacizumab continuation phase in some patients. The latter group was not included in the multivariate analysis.

On the other hand, the strengths of our work are the biological plausibility of the selected genes, the low number of genes included in the models, and the statistical method that offers a robust internal validation.

The G-model consists of 11 genes, and the GC-model includes 5 clinical covariates and 13 genes. Among the genes, 8 are present in both models: *SLC39A6, REL, IL8; FN1, PLAU, HES1, HMBS, DDIT4, FABP5, ACVRL1,* and *PGR*. The sign of beta coefficients agrees in all cases with the expression results of the univariate analysis of the genes, and is maintained in both models. *SLC39A6* and *REL* in the G-model, and *FN1* and, again, *REL* in the GC-model had the highest weight.

SLC39A6 expression has been described in clinical breast-tumour populations as significantly associated with the estrogen receptor status [15]. It has also been associated with the spread of breast cancer to regional lymph nodes [16, 17]. Interestingly, this gene had the highest beta coefficient in the G-model, but one of the lowest in the GC one, where ER is also included.

*FN1* has been previously described as part of an extracellular matrix gene cluster associated with resistance to first-line tamoxifen therapy in patients with mBC and with the development of metastasis [18] [19].

*REL* is part of the nuclear factor-κappaB (*NF-κB*) complexes. It had the highest beta coefficient in both models, being associated with improved PFS and OS. The involvement of *NF-κB* in neoplastic proliferation of human breast cancer cells has been described under estrogen-free conditions *in vitro*, where it induces additive anticancer effects with tamoxifen [20]. *Its* specific role in antiangiogenic therapy response should be further characterized.

We used LASSO Cox regression model to determine genes significantly related to PFS. This model accounts for overfitting, a problem inherent to studies where the number of genes far exceeds the number of patients. The output is a profile with a reduced number of genes, which makes it more suitable for clinical application. We also used LOOCV method, and a permutation test to assess the statistical significance of the models. Both GC and G models fared better than C model, being correlated with PFS and OS (Table 7).

We also tested the predictive accuracy of these models by time-dependent ROC curves (Figure 2). The relative weight of the gene component in G and GC models remained constant over time, whereas the clinical variables were important in the long term. A plausible explanation is that clinical parameters are purely prognostic, whereas genes predict benefit from treatment, so their influence is detected earlier.

In summary, we described two models that predict improved PFS and OS with bevacizumab-paclitaxel therapy: the G-model included a combination of 11 genes, and the (GC-model consisted of 13 genes and 5 clinical variables. Both had good accuracy in the first six months, whereas the GC-model remained accurate over time.

These findings should be evaluated in larger independent series in order to develop a routine clinical test to predict the benefit of bevacizumab in mBC patients.

## MATERIALS AND METHODS

### Patient selection and tumor sample collection

The study was approved by our institutional Ethics Committee. Patients with a diagnosis of HER2-negative mBC between 2007 and 2011 and treated with bevacizumab and paclitaxel were identified from local records. The schedule consisted of bevacizumab 10 mg/m2 days 1 and 15 plus weekly paclitaxel 80 mg/2 days 1, 8 and 15, on a 28-day cycle. REMARK criteria were used to help in patient selection [21].

Seventy eight patients fulfilled the inclusion criteria. Six of them were excluded due to the absence of primary tumor biopsy, and another 12 because of poor quality material. The study included the primary tumor from 60 patients. All of them had a minimum follow-up of 18 months since the beginning of the treatment (unless progression and/or death due to mBC occurred before). Patients with an early withdrawal due to toxicity were not included.

The variable selected to generate a gene signature was progression-free survival, defined as the time from the first administration of bevacizumab-paclitaxel treatment to the first evidence of relapse, death or last record available dates. Other outcome variables, such as response to treatment and overall survival, were also evaluated but were not used to develop the molecular signature.

### Gene expression analysis

#### RNA purification from FFPE samples

Sixty archival FFPE cases were evaluated by two breast cancer expert pathologists. Regions of invasive carcinoma were confirmed, and different areas with more than 80% of malignant epithelial cells were selected. Four to eight μm sections were used for total RNA isolation, with MasterPure RNA Purification Kit (EPICENTRE Biotechnologies, Madison, WI, USA) according to the manufacturer's instructions with minor modifications. RNA concentrations and quality were measured using a Nanodrop 1000A spectrophotometer (Nanodrop Technologies, Wilmington, DE, USA).

#### Real-Time Quantification of Gene Expression

One μg of total RNA was used for cDNA synthesis with the High Capacity Archive cDNA Reverse Transcription kit (Applied Biosystems, Foster City, CA, USA), and performed in an Applied Biosystems 7900 thermal cycler for 10 min at 25°C, 120 min at 37°C, and then held at 4°C.

The RT obtained products were amplified using a Real Time Ready Custom panel 384 (Hoffmann-La Roche, Basel, Switzerland). This platform enables the quantitation of 168 genes (Supplementary Table) plus 19 housekeeping (HK) genes and 5 internal controls per sample. The reactions were performed on the LightCycler^®^ 480 as follows: an initial step at 95°C for 10 min, 45 amplification cycles with 10s at 95°C, 30s at 60°C, and 1s at 72°C, and a final cooling step at 40°C for 30s.

#### Data processing

168 candidate genes were selected from the literature as related with the angiogenic process and other progression and resistance mechanisms, such as EMT or inflammation.

Nineteen housekeeping (HK) genes were also included in the study (Supplementary Table 1). Twelve of them were selected by GeNorm software [22] and used for the normalization factor applied to the raw data to get the relative expression value by the DDCt.

### Statistical analysis

Univariate Cox regression models were fitted to evaluate the association between continuous RNA expression values and clinical covariates with survival.

The prediction models were built with penalized multivariate Cox regression proportional hazards modelling using L1-penalized (Lasso). A cross-validated (CV) risk score was calculated by leave-one-out cross-validation (LOOCV) for each model [6, 7]. Patients were classified into high- and low-risk groups, based on the median value of the cross-validated risk score, and cross-validated Kaplan-Meier curves (CV KM) were estimated. A log-rank statistic was computed for the CV KM plots and the statistical significance was evaluated based on the permutation distribution of the cross-validated log-rank-statistic, repeating the whole LOOCV process with randomly permuted survival times and censoring indicators. Cross-validated time dependent receiver operating characteristics (ROC) curves were computed using the cross-validated risk scores. The area under the curve (AUC) values calculated from the CV ROC curves were used as measure of predictive accuracy of the models [8]. All statistical analyses were performed using R software (version 3.1.1) and the analysis was conducted by the *penalized* and *survival ROC* packages in R software.

### Inmunohistochemistry analysis

Genes found to be related to PFS in the multivariate analysis were further analyzed by tissue microarray. Representative areas of the tumors were selected on hematoxylin and eosin-stained sections and marked on individual paraffin blocks by an expert pathologist in the field. Two tissue cores (1 mm in diameter) were obtained from each specimen. The tissue cores were arrayed into a receptor paraffin block using a TMA workstation (Beecher Instruments, Silver Spring, MD, USA), as described previously [23]. A hematoxylin and eosin-stained section of the array was reviewed to confirm the presence of morphologically representative areas of the original lesions.

Immunohistochemistry (IHC) was performed as described previously [24] on 4um sections of the TMAs, obtained by a semiautomated microtome HM3508 (Microm). Briefly, the tissue sections were deparaffinized and rehydrated in water, after which antigen retrieval was carried out in a DAKO PT Link in citrate buffer (pH=6). Endogenous peroxidase and nonspecific antibody reactivity was blocked with peroxidase blocking reagent (Dako, Glostrup, Denmark) at room temperature for 15 min.

The sections were then incubated for 60 min with the following antibodies: rabbit monoclonal cMET (SP44, #790-4430, Hoffmann-La Roche Ltd, Basel, Switzerland) and cREL (EPR2258, #ab108299, Abcam, Cambridge, UK); rabbit polyclonal: FN1 (#A0245, Dako, Glostrup, Denmark), SLC39A6 (#ab61307), DDIT4 (#ab63059), and NOTCH3 (#ab60087), all these last from Abcam (Cambridge, UK). Detection was performed with Envision Plus Detection System (Dako, Glostrup, Denmark).

Cytoplasmic staining for cREL and SLC39A6, as well as nuclear and cytoplasmic staining for DDIT4 was classified as negative, focal or diffuse. NOTCH3 presence was diffuse when present, so we evaluated absence or weak/strong staining. Stromal FN1 staining observed in the stromal component was also evaluated. The pathologists performing the immunohistochemistry analysis were blinded for patient's outcome.

## SUPPLEMENTARY TABLE




