# Supplementary material for: Predictive value of angiogenesis-related gene profiling in patients with HER2-negative metastatic breast cancer treated with bevacizumab and weekly paclitaxel
Source: Oncotarget. 2016 Mar 16;7(17):24217–27. doi: 10.18632/oncotarget.8128 (PMC5029696; doi:10.18632/oncotarget.8128)
Supplement: Supplementary file 1 [file oncotarget-07-24217-s001.pdf]

SUPPLEMENTARY TABLE

Supplementary Table S1: Gene list, HR (95% IC) and p values for univariate analysis

See Supplementary File 1
