# Supplementary material for: Predictive value of angiogenesis-related gene profiling in patients with HER2-negative metastatic breast cancer treated with bevacizumab and weekly paclitaxel
Source: Oncotarget. 2016 Mar 16;7(17):24217–27. doi: 10.18632/oncotarget.8128 (PMC5029696; doi:10.18632/oncotarget.8128)
Supplement: Supplementary file 2 [file oncotarget-07-24217-s002.docx]

**Suppl. Table.** Gene list, HR (95% IC) and p values for univariate analysis.

| ***Gene Symbol*** | ***HR*** | ***P Value*** |  | ***Gene Symbol*** | ***HR*** | ***P Value*** |  | ***Gene Symbol*** | ***HR*** | ***P Value*** |
| --- | --- | --- | --- | --- | --- | --- | --- | --- | --- | --- |
| **ACVRL1** | 1.21 (0.99-1.48) | 0.0617 |  | **FGFR4** | 0.97 (0.86-1.09) | 0.5846 |  | **NRP1** | 0.89 (0.64-1.22) | 0.4678 |
| **ADM** | 0.96 (0.87-1.07) | 0.4878 |  | **FIGF** | 1.01 (0.91-1.13) | 0.8144 |  | **NRP2** | 0.97 (0.85-1.11) | 0.6846 |
| **ADRBK1** | 0.95 (0.77-1.17) | 0.6332 |  | **FLT1** | 1 (0.86-1.17) | 0.9826 |  | **OCLN** | 1.09 (0.97-1.24) | 0.1622 |
| **ALDH4A1** | 1.1 (0.92-1.31) | 0.2705 |  | **FLT4** | 1.03 (0.9-1.19) | 0.6351 |  | **PARK7** | 1.04 (0.75-1.45) | 0.8115 |
| **ANGPT1** | 1.01 (0.87-1.18) | 0.8485 |  | **FLVCR2** | 1 (0.89-1.11) | 0.9546 |  | **PAX8** | 1.01 (0.9-1.13) | 0.903 |
| **ANGPT2** | 1 (0.89-1.13) | 0.9869 |  | **FN1** | 1.27 (1.1-1.47) | 0.0026* |  | **PDGFRA** | 0.95 (0.84-1.09) | 0.505 |
| **ANGPT4** | 1.01 (0.9-1.13) | 0.903 |  | **FOXC2** | 0.98 (0.9-1.07) | 0.6776 |  | **PDGFRB** | 1.19 (0.97-1.46) | 0.0948 |
| **ANGPTL4** | 0.98 (0.87-1.09) | 0.6662 |  | **FYN** | 0.97 (0.83-1.14) | 0.7353 |  | **PDK1** | 0.96 (0.85-1.08) | 0.4904 |
| **AURKA** | 0.95 (0.85-1.06) | 0.3653 |  | **GABPA** | 0.96 (0.8-1.15) | 0.6729 |  | **PGR** | 1.13 (1.03-1.25) | 0.0118* |
| **AURKB** | 0.99 (0.89-1.11) | 0.8723 |  | **GAL** | 0.94 (0.84-1.05) | 0.2743 |  | **PLAU** | 1.24 (1.07-1.44) | 0.0052* |
| **AURKC** | 1 (0.9-1.12) | 0.9534 |  | **GRB7** | 1.02 (0.91-1.16) | 0.6933 |  | **PLOD1** | 0.94 (0.71-1.25) | 0.6791 |
| **BAG1** | 1.09 (0.93-1.27) | 0.2837 |  | **GSTM1** | 0.98 (0.89-1.08) | 0.6916 |  | **PPBP** | 1.03 (0.92-1.15) | 0.6461 |
| **BCL2** | 0.96 (0.85-1.08) | 0.4954 |  | **HBB** | 1.03 (0.9-1.17) | 0.7073 |  | **PTGS2** | 0.95 (0.84-1.08) | 0.4505 |
| **BIRC5** | 1.06 (0.89-1.25) | 0.5197 |  | **HELT** | 0.98 (0.9-1.06) | 0.5646 |  | **PTK2B** | 1.12 (0.96-1.3) | 0.1341 |
| **BMI1** | 1.04 (0.87-1.24) | 0.6737 |  | **HES1** | 1.07 (0.96-1.21) | 0.2635 |  | **PTK6** | 0.98 (0.86-1.11) | 0.7709 |
| **BMP1** | 0.99 (0.87-1.14) | 0.9077 |  | **HIF1A** | 1 (0.89-1.13) | 0.9873 |  | **PTPRC** | 1 (0.91-1.1) | 0.9745 |
| **BMP2** | 1.01 (0.9-1.13) | 0.9134 |  | **HMBS** | 0.9 (0.8-1.02) | 0.1014 |  | **REL** | 0.88 (0.77-1) | 0.0619 |
| **BMP4** | 1.01 (0.9-1.14) | 0.8367 |  | **HOXB13** | 1.03 (0.92-1.16) | 0.5867 |  | **ROBO1** | 0.99 (0.88-1.11) | 0.8945 |
| **BMP7** | 0.97 (0.87-1.09) | 0.6641 |  | **ID1** | 0.94 (0.79-1.1) | 0.4344 |  | **ROBO4** | 1.02 (0.9-1.15) | 0.7727 |
| **BSG** | 1.05 (0.85-1.3) | 0.6637 |  | **ID2** | 1.18 (0.9-1.54) | 0.2211 |  | **RRAGD** | 0.97 (0.84-1.12) | 0.6378 |
| **BST1** | 1.02 (0.9-1.16) | 0.7441 |  | **ID3** | 1.03 (0.89-1.18) | 0.727 |  | **RUNX1** | 0.95 (0.82-1.1) | 0.4997 |
| **CA9** | 0.98 (0.87-1.09) | 0.6669 |  | **ID4** | 1 (0.9-1.12) | 0.9523 |  | **S1PR1** | 0.96 (0.87-1.06) | 0.4322 |
| **CAV1** | 1 (0.84-1.18) | 0.9846 |  | **IGF1R** | 1.11 (0.95-1.31) | 0.1824 |  | **SERPINB2** | 1 (0.89-1.12) | 0.9814 |
| **CAV2** | 0.96 (0.82-1.13) | 0.6377 |  | **IGFBP2** | 1.09 (0.96-1.23) | 0.1999 |  | **SERPINE1** | 1.02 (0.86-1.21) | 0.8334 |
| **CCL2** | 0.99 (0.82-1.2) | 0.9319 |  | **IGFBP3** | 0.99 (0.88-1.11) | 0.8571 |  | **SHH** | 1.01 (0.9-1.13) | 0.903 |
| **CD24** | 0.95 (0.81-1.1) | 0.4884 |  | **IHH** | 0.95 (0.87-1.04) | 0.2676 |  | **SLC16A1** | 0.92 (0.77-1.11) | 0.3945 |
| **CD36** | 1 (0.88-1.12) | 0.9346 |  | **IL13** | 1.01 (0.9-1.13) | 0.903 |  | **SLC16A3** | 1 (0.86-1.16) | 0.9933 |
| **CD44** | 1.09 (0.96-1.25) | 0.1967 |  | **IL17RB** | 1.04 (0.96-1.14) | 0.3337 |  | **SLC16A4** | 1.06 (0.92-1.23) | 0.3903 |
| **CD47** | 0.95 (0.81-1.11) | 0.5501 |  | **IL23A** | 1 (0.89-1.13) | 0.9867 |  | **SLC2A1** | 1 (0.83-1.19) | 0.9732 |
| **CD68** | 0.96 (0.79-1.16) | 0.6854 |  | **IL6** | 0.98 (0.88-1.09) | 0.7271 |  | **SLC39A6** | 1.31 (1.13-1.52) | 4.00E-04* |
| **CDH11** | 1.28 (1.05-1.57) | 0.0153* |  | **IL8** | 0.92 (0.86-0.97) | 0.0053* |  | **SPARC** | 1.16 (0.88-1.53) | 0.3021 |
| **CDH13** | 1.03 (0.9-1.19) | 0.6395 |  | **ITGB2** | 0.92 (0.76-1.13) | 0.4335 |  | **SPP1** | 1.01 (0.89-1.15) | 0.8859 |
| **CDH5** | 1.07 (0.89-1.28) | 0.4662 |  | **JAG1** | 1 (0.87-1.14) | 0.9471 |  | **STAT3** | 1.06 (0.87-1.28) | 0.5594 |
| **CLDN4** | 1.05 (0.87-1.26) | 0.6036 |  | **JAG2** | 1.03 (0.91-1.16) | 0.683 |  | **TCF3** | 0.94 (0.77-1.14) | 0.5341 |
| **CSF2** | 1.01 (0.9-1.13) | 0.903 |  | **JAM2** | 1.04 (0.89-1.22) | 0.6098 |  | **TEK** | 1 (0.88-1.14) | 0.9731 |
| **CSK** | 1.04 (0.86-1.25) | 0.7178 |  | **JAM3** | 1.06 (0.88-1.27) | 0.5503 |  | **TGFB1** | 1.21 (0.99-1.49) | 0.0861 |
| **CTNNB1** | 1.09 (0.92-1.29) | 0.2934 |  | **KDR** | 0.99 (0.84-1.15) | 0.8554 |  | **TGFB2** | 1.07 (0.93-1.22) | 0.3488 |
| **CTSL2** | 1 (0.88-1.13) | 0.989 |  | **KIT** | 1 (0.88-1.13) | 0.9616 |  | **TGFB3** | 1.17 (0.99-1.37) | 0.0652 |
| **CXCL1** | 0.95 (0.85-1.06) | 0.3417 |  | **KLF4** | 1.01 (0.9-1.13) | 0.8911 |  | **TGFBR1** | 0.97 (0.77-1.23) | 0.8179 |
| **CXCL12** | 1.27 (0.95-1.7) | 0.1008 |  | **LOX** | 1.03 (0.88-1.21) | 0.7105 |  | **TGFBR2** | 0.98 (0.81-1.17) | 0.7959 |
| **CXCL2** | 1.01 (0.9-1.13) | 0.9044 |  | **LOXL2** | 1.03 (0.89-1.19) | 0.7061 |  | **THBS1** | 1.09 (0.91-1.31) | 0.3683 |
| **DDIT4** | 1.07 (0.93-1.22) | 0.3471 |  | **MAML1** | 1.14 (0.93-1.4) | 0.2281 |  | **TIE1** | 1.04 (0.91-1.18) | 0.5923 |
| ***Gene Symbol*** | ***HR*** | ***P Value*** |  | ***Gene Symbol*** | ***HR*** | ***P Value*** |  | ***Gene Symbol*** | ***HR*** | ***P Value*** |
| **DLG1** | 1.23 (0.96-1.58) | 0.1168 |  | **MAML2** | 0.92 (0.79-1.06) | 0.2408 |  | **TIMP1** | 1.15 (0.92-1.45) | 0.2398 |
| **DLL4** | 1.03 (0.89-1.18) | 0.7043 |  | **MAML3** | 1.05 (0.91-1.23) | 0.4866 |  | **TJP1** | 1.24 (0.93-1.64) | 0.1326 |
| **EDN1** | 0.99 (0.87-1.13) | 0.8645 |  | **MBTPS1** | 1.01 (0.86-1.18) | 0.9343 |  | **TNFRSF11A** | 0.96 (0.84-1.09) | 0.5328 |
| **EGFR** | 0.98 (0.85-1.12) | 0.7286 |  | **MET** | 1.01 (0.89-1.15) | 0.8956 |  | **TNFSF11** | 0.98 (0.88-1.08) | 0.6631 |
| **EGLN1** | 0.99 (0.89-1.11) | 0.8845 |  | **MKI67** | 1.06 (0.91-1.23) | 0.4517 |  | **TP63** | 1 (0.89-1.12) | 0.969 |
| **EPAS1** | 1 (0.89-1.13) | 0.9936 |  | **MMP11** | 1.1 (0.99-1.23) | 0.0653 |  | **TWIST1** | 0.96 (0.87-1.04) | 0.3157 |
| **EPCAM** | 1.13 (0.95-1.34) | 0.2028 |  | **MUC1** | 1.11 (0.97-1.26) | 0.132 |  | **TWIST2** | 0.95 (0.84-1.08) | 0.4581 |
| **EPHA2** | 0.93 (0.82-1.05) | 0.2606 |  | **MYBL2** | 1.01 (0.9-1.13) | 0.8986 |  | **UCHL1** | 1.01 (0.9-1.13) | 0.903 |
| **ERBB2** | 1.06 (0.9-1.26) | 0.4515 |  | **NCAM1** | 1 (0.89-1.12) | 0.9654 |  | **VEGFA** | 0.91 (0.75-1.12) | 0.3619 |
| **ESR1** | 1.12 (1.02-1.24) | 0.0202* |  | **NDRG1** | 0.84 (0.67-1.05) | 0.1231 |  | **VEGFC** | 1.09 (0.94-1.27) | 0.2352 |
| **F11R** | 1.17 (0.95-1.44) | 0.1302* |  | **NODAL** | 1.01 (0.9-1.13) | 0.903 |  | **VHL** | 1.04 (0.8-1.36) | 0.77 |
| **FABP5** | 0.86 (0.75-0.98) | 0.0245* |  | **NOTCH1** | 1.02 (0.88-1.18) | 0.81 |  | **VIM** | 1.06 (0.78-1.44) | 0.7067 |
| **FGF1** | 1.07 (0.93-1.23) | 0.3455 |  | **NOTCH2** | 1.01 (0.82-1.24) | 0.9325 |  | **ZEB1** | 0.96 (0.83-1.11) | 0.6036 |
| **FGF2** | 0.92 (0.84-1.02) | 0.1259 |  | **NOTCH3** | 1.4 (1.03-1.89) | 0.0293* |  | **ZEB2** | 0.93 (0.79-1.1) | 0.3926 |
| **FGFR1** | 0.98 (0.83-1.17) | 0.8368 |  | **NOTCH4** | 0.98 (0.87-1.11) | 0.7451 |  |  |  |  |
